# Case report and literature review: Asymptomatic littoral cell angioma in a 3-year-old girl

**DOI:** 10.3389/fped.2024.1383015

**Published:** 2024-04-18

**Authors:** Yanling Mou, Liucheng Yang, Jianjun Wang, Qinming Chen, Mengzhen Zhang, Xi Zhang, Rongying Tan, Djibril Adam Mahamat, Kai Wu

**Affiliations:** Department of Pediatric Surgery, Zhujiang Hospital, Southern Medical University, Guangzhou, Guangdong, China

**Keywords:** littoral cell angioma, splenic tumor, children, treatment, diagnosis

## Abstract

**Background:**

Littoral cell angioma (LCA) is an extremely uncommon benign vascular tumor of the spleen. Cases of LCA in infants are rarely reported, and due to the rarity of the tumor and non-specific symptoms, the diagnosis of LCA is often overlooked in clinical practice.

**Case report:**

We present a 3-year-old girl with pulmonary inflammation who was admitted to the hospital due to the discovery of a space-occupying lesion in the spleen. Pathology after splenectomy confirmed LCA, and there was no recurrence observed at the 5-month follow-up examination.

**Conclusion:**

LCA should be considered when a child shows asymptomatic splenomegaly, with antigen expression indicating dual positivity of endothelial and histiocytic markers. Laparoscopic splenectomy remains the primary method of treating LCA.

## 1 Introduction

Primary splenic tumors in children are clinically rare, accounting for only 0.03% of all tumors. Splenic tumors can be classified as benign and malignant, with the majority of benign cases including hemangiomas, lymphangiomas, and splenic cysts. In contrast, malignant tumors include lymphoma, leiomyosarcoma, and others. Splenic tumors are usually asymptomatic, with 50% of patients accompanied by splenic enlargement or hypersplenism, leading to anemia or pancytopenia (1). Therefore, its diagnosis is usually incidental.

Littoral cell angioma (LCA) is a rare occurrence in splenic tumors, arising from normal spleen red sinus shore cells and belonging to the reticuloendothelial cell system. Tumor cells exhibit the specific characteristics of double differentiation, including both endothelial and histiocyte antigens (1). Unlike general hemangiomas, LCA has the potential for recurrence and metastasis, necessitating timely surgical intervention. Since LCA tends to strike in middle age, reports of LCA in children are extremely rare. Herein, we present the diagnosis and treatment experience of LCA in a 3-year-old girl, and conduct a literature review of LCA in children, aiming to provide references for the clinical diagnosis and treatment of pediatric surgeons.

## 2 Case report

**Figure 1:**
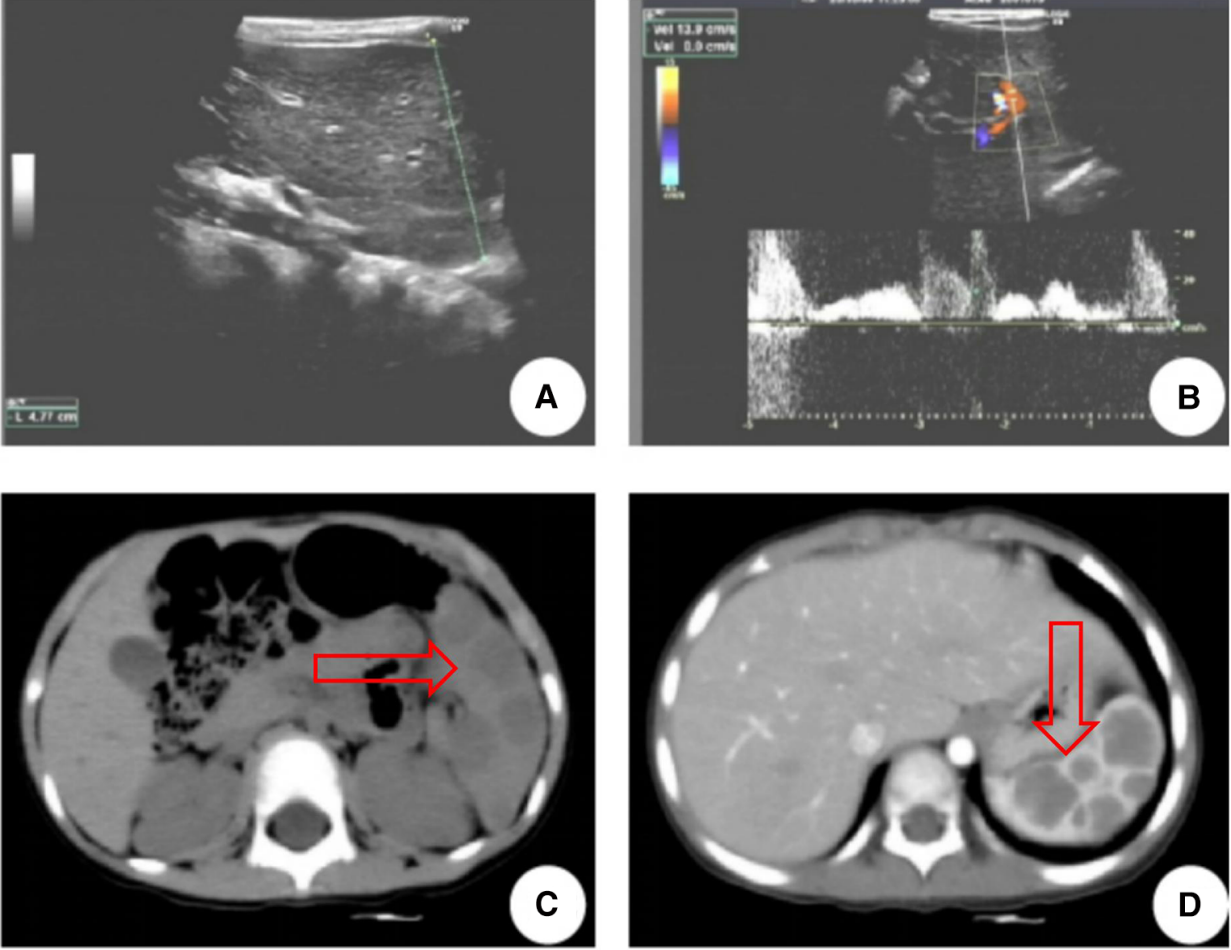
LCA patient's ultrasound and CT images. (**A**,**B**) Ultrasound indicated multiple solid slightly hyperechoic masses were diffusely distributed in the spleen, measuring approximately 26 mm × 23 mm, 12 mm × 13 mm, 19 mm × 19 mm, 9 mm × 9 mm, 8 mm × 5 mm, etc. (**C**) CT revealed multiple nodular lesions in the spleen. (**D**) Enhancement reveals multiple masses of varying sizes within the spleen, with uneven internal enhancement and weaker than the spleen. The arrows point to the tumor.

**Figure 2:**
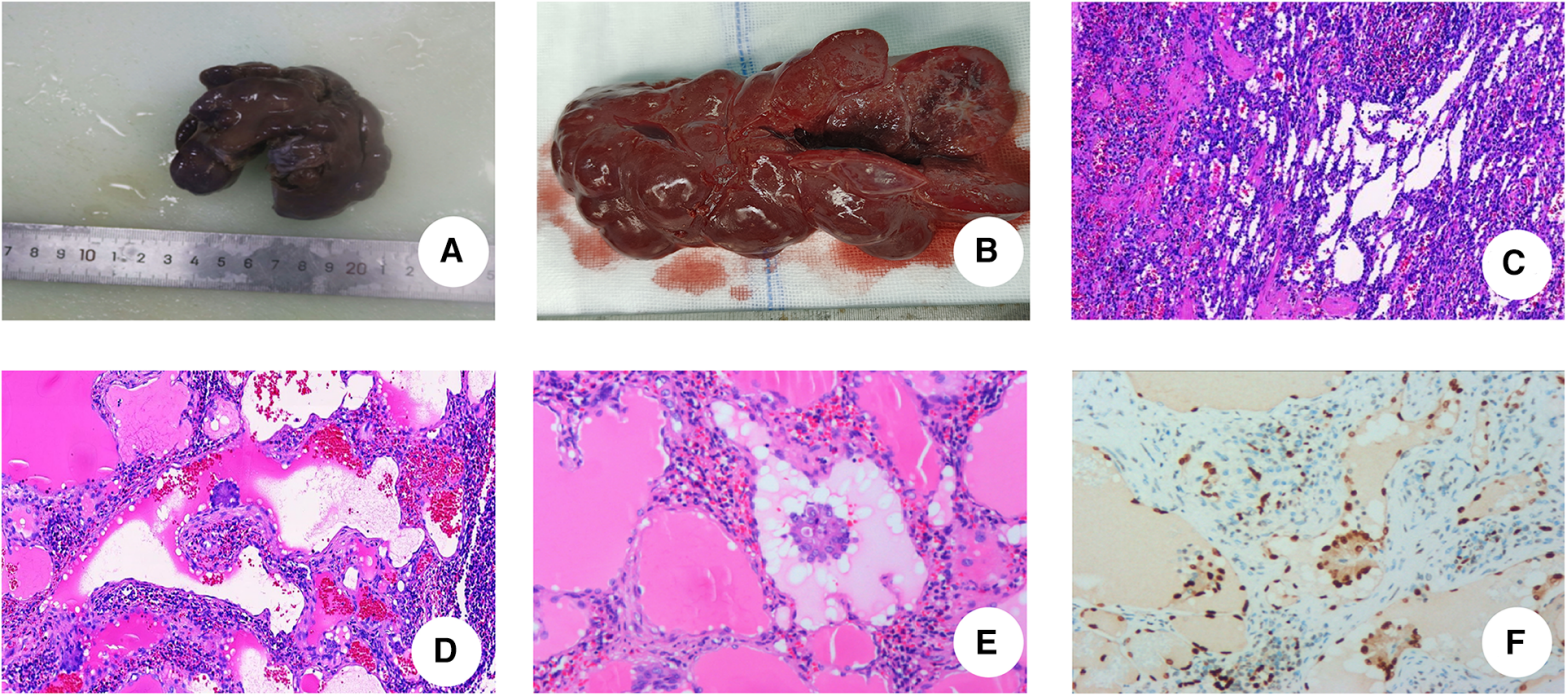
Postoperative pathological examinations. (**A**) The spleen significantly enlarged, with measured 10.5 cm × 5 cm × 3 cm. (**B**) The spleen is grayish-red, with nodular cut surfaces, solid, and slightly firm in consistency. (**C**) Hematoxylin and eosin staining of LCA. The tumor consists of an interlacing vascular network similar to splenic sinus, with papillary projections and cystic cavities (IHC × 5). (**D**) Papillary structures are seen in the lumen of the blood vessels (IHC × 10). (**E**) Nuclei with no obvious heterogeneity. (IHC × 20). (**F**) ERG staining: ERG(+) (IHC × 20).

A 3-year-old girl had originally sought treatment at the local hospital 5 months earlier for persistent coughing. A computed tomography (CT) of the abdomen revealed splenomegaly with multiple nodules. Upon physical examination, mild tenderness in the upper left abdomen was noted, along with a palpable spleen enlargement located approximately 5 cm below the costal margin. The spleen exhibited a smooth surface and firm texture. Blood routine was as follows: white blood cells 5.38 × 10^9^/L, red blood cells 3.89 × 10^9^/L, hemoglobin 107 g/L, and platelets 179 × 10^9^/L. An ultrasound examination indicated irregular splenic enlargement with uneven internal echoes. Multiple solid slightly hyperechoic masses were diffusely distributed in the spleen. In order to further clarify the mass and vascular distribution, a CT scan was performed, revealing an enlarged spleen with multiple variably sized mass-like and nodular low-density lesions. The large lesion, measuring approximately 44 mm × 23 mm, which was located at the lower pole of the spleen, exhibited uniform density and enhancement on contrast-enhanced scans. Some lesions exhibited obvious enhancement with indistinct edges and were surrounded by splenic vascular branches (Figure 1). Thus, hereditary spherocytosis and portal hypertension (PHT) were excluded as potential causes for splenomegaly. Given the girl's prolonged consumption of raw water, serological testing revealed positive antibodies against *Leptospira* and *Angiostrongylus cantonensis*. Additionally, consecutive stool examinations were conducted on three separate occasions, all yielding negative results. To further rule out the association with parasites, the patient underwent magnetic resonance imaging (MRI) of the brain, which revealed there were no space-occupying lesions in the brain. Eventually, the space-occupying lesion in the spleen was considered to be a tumor. A laparoscopic splenectomy was then performed. The pathological examination showed multiple solid nodules of varying sizes evident in the cross-section of the spleen. Broadly speaking, the microscopic picture showed a multifocal tumor. Specifically, the tumor was located within the red pulp and was well demarcated from the surrounding normal tissue without necrosis or hemorrhage. Microscopically, the tumor consisted of vascular lumens of varying sizes, lined with blood vessels and a single layer of proliferating endothelial cells. Papillary-like structures were evident, and the lumens contained decidual cells. The cells were large, with abundant cytoplasm, and iron-containing hemosiderin granules were observed in the cytoplasm. Tumor cells are homogeneous, and nuclear division is rare (Figure 2). Immunohistochemical (IHC) analysis of the tumor cells showed positive staining for vimentin/CD31, ERG, F8, CD34 (partly), with focally positive for CD68, CD163, and D2-40; Ki-67 was approximately 2%. These findings confirmed the diagnosis of LCA. Five months postoperatively, outpatient ultrasonography revealed no tumor residue or recurrence.

## 3 Discussion

LCA is a relatively uncommon benign tumor of the spleen, primarily affecting individuals aged 40–60 years (2). It is notably scarce in the pediatric population, with the existing literature predominantly consisting of case reports. Since its initial identification by Falk et al. (1) in 1991, 11 cases have been reported in children younger than 16 years old (Table 1). The cohort comprised seven boys and five girls (age range 26 days–15 years) who were incidentally diagnosed due to splenomegaly and hyperfunction of the spleen.

**Table 1:** Clinical data of children with LCA.

Authors	Age	Sex	Clinical presentation	Routine blood test	Imaging results	Tumor types	Biomarkers	Treatment and prognosis
Antón-Pacheco et al. (3)	1	F	Splenomegaly, fever	Moderate anemia, decreased PLT	MRI: high density	Multiple	Factor VIII^+^, CD31^+^, CD34^+^, CD68^+^ Phagocytosis and hemosiderin deposition	Splenectomy No recurrence/5 year
Ertan et al. (4)	2	M	Severe splenomegaly	Severe anemia, low fibrinogen, decreased PLT	CT: low-density shadows in arterial phase MRI: low signal on T2 and high signal on T3 Ultrasound: low-density shadows	Multiple	—	Splenectomy
Bedir et al. (5)	11	F	Abdominal pain	PLT, Hb both normal	MRI: low signal on T1, high signal on T2	Solitary	CD31^+^, CD68^+^, CD34^−^, CD8^−^, Ki67 (2%)	Splenectomy
Forest et al. (6)	12	M	Splenomegaly	—	Ultrasound: low-density	Multiple	Hemosiderin deposition and phagocytosis	Splenectomy
Li et al. (7)	15	M	Abdominal pain	PLT, Hb both normal	Enhanced CT: high density in early arterial phase, low density in portal venous phase	Solitary	—	Splenectomy
Gakenheimer-Smith et al. (8)	26 days	M	Vomiting with abdominal distension	Decreased PLT	CT: low echo MRI: decreased T1 signal intensity, increased T2 signal intensity	Multiple	ERG^+^, CD68^+^ and CD21^+^, CD8^−^, CD34^−^, and WT1^−^	Conservative No recurrence/7 months
Shah et al. (9)	2	M	Recurrent nosebleeds and multiple purpura, splenomegaly	Moderate anemia	—	—	—	Splenectomy No recurrence/6 years
Wu (10)	7	F	None	PLT, RBC both normal	MRI: slightly long T1, isointense T2 DWI slightly low signal, central radial short T2 signal	Solitary	CD31^+^, CD34^+^, D2-40^+^, Ki67^+^	Splenectomy No recurrence/21 months
Anbardar et al. (11)	11	M	Anemia, abdominal pain and bloating, splenomegaly	Mild anemia, mild decrease in PLT	Ultrasound: low echoes	Multiple	CD68^+^, CD31^+^, CD34^+^ Phagocytosis and hemosiderin pigmentation	Splenectomy
Wang et al. (12)	9	F	Abdominal pain	—	CT: mixed density of the spleen, central patchy high-density Ultrasound: heterogeneous echoes	Solitary	CD31^+^, CD34^−^, ERG^+^, EMA^+^, Vim^−^, Ki-67^+^	Splenectomy
Lai et al. (13)	9	M	Occasionally abdominal pain, splenomegaly	—	MRI: equal/slightly prolonged T1 and T2	Solitary	CD31^+^, CD34 ^+^, CK^−^, Ki-67 ^+^, Vimentin^+^, AATC^+^, CD68^+^	—
Our case	3	F	None	Mild anemia	CT: low-density Ultrasound: slightly stronger echoic	Multiple	Vimentin^+^/CD31^+^, ERG^+^, F8^+^, CD34^+^, CD68^+^, CD163^+^, CD21^−^, D2-40^+^, CK^−^, Ki67^+^	Splenectomy No recurrence/5 months

LCA was categorized as a type of spleen-specific vascular interstitial tumor (14). The exact pathogenetic mechanisms responsible for LCA are unknown but dysregulation of the immune system is likely. Li et al. (15) revealed that one-third of their cases were accompanied by visceral malignancies, whereas another third of their patients suffered from immune system diseases or immune dysfunctions. Due to the characteristics of the pediatric group, there were fewer tumors among the accompanying diseases in the pediatric patients with LCA. Occasionally, LCA occurs in children due to immune dysfunction caused by congenital diseases of the immune system or immunosuppressive treatment (16). In addition, as with other diseases, there appears to be a correlation between LCA and the inflammatory factor storm (17). Given occasional individual reports of siblings in a family having both LCA and littoral cell angiosarcoma (LCAS) (18), this disease may have genetic susceptibility or familial clustering. Notably, a pediatric patient with LCA also had Gaucher's disease, an autosomal recessive genetic disorder, which may also support the possibility of the genetic connection in pediatric cases of LCA. Interestingly, not all researchers agree that LCA is composed of tumor cells. After reviewing 17 cases of LCA pathology, Bi et al. (19) proposed that proliferating endothelial cells are a benign granulomatous response triggered by circulating tumor cell-immune cell complexes during the blood-filtering process in the splenic sinuses.

Imaging examinations hold certain diagnostic significance for this condition, although they lack specificity. CT scans typically reveal low-density splenic masses, marked by delayed enhancement. These characteristics were observed in 10 children with LCA. MRI can show a distinctive speckled sign, the extent of which depends on the amount of hemosiderin in the tumor's endothelial cells. Due to the paramagnetic effect of iron, the increased deposition of hemosiderin within cells can reduce the signal on T2-weighted imaging (T2WI). This characteristic aids in differentiating LCA from other vascular tumors and serves as a specific manifestation of LCA (20). In pediatric cases, MRI was performed in six cases, with five cases showing high signal intensity in the T2 phase of the lesion, displaying uneven internal signals mixed with speckled low signals, known as the speckled sign.

Currently, there are no standard diagnostic guidelines for LCA, as it lacks specific clinical symptoms and imaging features. The definitive diagnosis primarily relies on pathological examination. LCA usually presents multiple lesions in the spleen, whereas in children, it exhibits varied of types. Our review revealed an equal occurrence of single and multiple lesions, comprising five cases of multiple nodular lesions, five cases of solitary nodular lesions, and one case with an unspecified type of lesion. Even though the main biological behavior of LCA is benign, caution must be taken due to its close relationship with malignant masses. Malignant transformation occurs when the spleen weighs more than 1,500 g or its diameter exceeds 20 cm (21). Compared to normal sinus endothelial cells, a littoral cell is characterized by dual antigen expression, with both endothelial and histiocytic markers being positive, occasionally showing positivity for transferrin receptors, distinguishing it from hemangiomas. IHC shows a positive staining for endothelial markers [such as Factor VIII (FVIII), CD31, von Willebrand Factor (vWF), CD34] and histiocytic markers (CD 68) (1). Although patients with LCA generally do not express CD34, recent studies have suggested the possibility of LCA malignancy in cases where CD34 is positive (22). In this cohort of children, CD68 was positive in five cases. The patient in our hospital showed dual differentiation in immunohistochemical staining results, with Ki-67 positivity (approximately 2%), and pathology revealed phagocytosis and hemosiderin pigment deposition, consistent with previous reports. The primary components of the differential diagnosis include lymphomas, tumors of vascular origin, and LCAS, which appear to be easier to distinguish than the former two because of the markedly heterogeneous nature of its cells, active nuclear division, and frequent necrosis. In fact, LCA uniquely exhibits as double antibody-positive to distinguish it from the vast majority of diseases, which is the basis of the pathological diagnosis for LCA.

Surgeons often grapple with fundamental questions related to the management of LCA. When a splenic mass is discovered, it is crucial to accurately determine the degree of benignity or malignancy of the tumor. If there is a potential for malignancy within a benign mass, a rational treatment plan should be selected. In the pediatric population in particular, the spleen functions as an immune organ. Explaining the need for splenectomy to guardians and taking measures to prevent and manage post-splenectomy infections (OPS) play essential roles. Surgeons should determine the most beneficial treatment for their patients through a careful evaluation process. Here, some evidence suggests that conservative treatment has no potentially malignant effects on patients (8). While Li et al. suggested close monitoring (12), others advocated radical splenectomy instead, considering that LCA poses potential risks of malignancy, recurrence, and metastasis (23). Susan et al. suggested that progressive splenic enlargement can also lead to a decrease in blood cells and even rupture, causing catastrophic hemorrhage. Eventually, after discussions with our medical team, splenectomy was cautiously recommended, considering that malignant transformation can be easily overlooked. Methods of splenectomy include laparoscopic splenectomy and splenectomy. Nowadays, total splenectomy under laparoscopy is generally carried out due to its advantages of being safe, effective, and minimally invasive (24, 25). In fact, the final choice for total or partial splenectomy depends on the patient's age and the size of the splenic tumor; partial splenectomy can be performed when there is a small, limited lesion within the spleen (16). Surgery is not the only treatment for the disease, and medication such as etoposide, paclitaxel, and vincristine may sometimes play a role (26). Although chemotherapy showed a significant anti-tumor effect, it is still not the main treatment for LCA. In all cases of children with LCA, splenectomy was performed in most cases, and only one case received conservative treatment. No recurrence was observed in all cases during follow-up.

Following pediatric splenectomy guidelines (27), the patient received a prompt vaccination with a triple vaccine upon discharge. In addition, a long-term anticoagulation therapy was initiated consisting of warfarin and regular injections of long-acting penicillin. Notably, no recurrence was observed during the 5-month follow-up period.

## Data Availability

The original contributions presented in the study are included in the article/Supplementary Material, further inquiries can be directed to the corresponding author.
